# Association of disability and 12-year all-cause and cause-specific mortality: analyses from the Wellbeing of Older People cohort study in Uganda

**DOI:** 10.1136/bmjgh-2025-019802

**Published:** 2026-01-23

**Authors:** Ivan Kasamba, Joseph Mugisha, Sarah Marks, Pelegrino Mbabazi, Femke Bannink-Mbazzi, Janet Seeley, Hannah Kuper

**Affiliations:** 1 Medical Research Council/Uganda Virus Research Institute and London School of Hygiene & Tropical Medicine Uganda Research Unit, Entebbe, Uganda; 2 Department of Infectious Disease Epidemiology and International Health, London School of Hygiene & Tropical Medicine, London, UK; 3 International Centre for Evidence in Disability, London School of Hygiene & Tropical Medicine, London, UK; 4 Department of Global Health and Development, London School of Hygiene & Tropical Medicine, London, UK

**Keywords:** Cohort study, Universal Health Care, Aging

## Abstract

**Introduction:**

Disability is linked to poor health outcomes and increased mortality, yet evidence on this relationship in sub-Saharan Africa is limited. This study investigated the association between disability and all-cause and cause-specific mortality among older adults in Uganda.

**Methods:**

The analysis was based on longitudinal data from the Wellbeing of Older People Study, an open cohort of individuals aged at least 50 years and followed over five data collection waves from 2009 to 2022. Disability was assessed using the WHO Disability Assessment Schedule 2.0. Mortality data were collected, supplemented by verbal autopsies. Gompertz regression models examined the association between disability severity and mortality, adjusting for sociodemographic, socioeconomic, health access and health risk factors.

**Results:**

Among 938 participants followed up for a median of 8.0 years (interquartile range (IQR): 3.2–11.5), 153 deaths were recorded (mortality rate: 2.4 per 100 person-years). The age-sex-adjusted analyses showed that the hazard ratio (HR) was 3.88-fold higher (95% confidence interval (CI) 2.50 to 6.02; p value <0.001) among people with severe disability compared with none/mild disability. Adjusting for sociodemographic, economic, social support and health factors (health status, access and risk factors) somewhat attenuated the association (adjusted HR (aHR) 3.08, 95% CI 1.92 to 4.93; p value <0.001). This excess risk persisted across broadly categorised causes of death: HIV (aHR 8.96, 95% CI 2.52 to 31.83), communicable diseases excluding HIV (aHR 2.24, 95% CI 0.80 to 6.25), non-communicable diseases (aHR 2.29, 95% CI: 1.20 to 4.37) and indeterminate causes of death (aHR 6.89, 95% CI 1.63 to 29.1). Additionally, disability was associated with sociodemographic disadvantages, poor healthcare access and higher prevalence of health risk factors.

**Conclusions:**

Severe disability was strongly associated with elevated mortality risk among older Ugandans, underscoring the need for targeted interventions to improve health equity. Reducing mortality disparities might require addressing barriers to healthcare access, stronger social support and integrating disability-inclusive policies in achieving global health targets, including Universal Health Coverage.

**WHAT IS ALREADY KNOWN ON THIS TOPIC**

- Disability is associated with poor health outcomes and increased mortality risk globally in part due to barriers in healthcare access for people with disabilities. Most of the evidence on the relationship between disability and mortality comes from high-income settings, with limited data from sub-Saharan Africa.

**WHAT THIS STUDY ADDS**

- This study provides one of the first longitudinal analyses of the relationship between disability and mortality in sub-Saharan Africa, focusing on older adults in Uganda. It demonstrates a threefold higher risk of all-cause mortality among individuals with severe disability compared with those with no/mild disability—independent of sociodemographic, socioeconomic and health-related factors. The study also shows that people with disabilities consistently had excess mortality risk across broad categories for cause of death: HIV, communicable diseases excluding HIV and non-communicable diseases.

**HOW THIS STUDY MIGHT AFFECT RESEARCH, PRACTICE OR POLICY**

- These study findings underscore the need for further investigation into the drivers of health inequities among people with disabilities in Uganda and sub-Saharan Africa, as well as developing and testing interventions to reduce mortality risk in this population. The findings also underline the need to address barriers to healthcare access, strengthen social support and disability-inclusive policies.

## Introduction

 Disability often adversely affects individuals’ health and well-being, potentially increasing the risk of mortality.^1^,^4^ First, the impairment or health condition underlying the disability (eg, Down syndrome or visual impairment) may increase the risk of further health conditions either directly (eg, Down syndrome elevates risk of leukaemia) or indirectly (eg, wheelchair users are at risk of pressure sores). Second, people with disabilities frequently experience poverty and marginalisation, a higher prevalence of disease risk factors (eg, smoking) and barriers when accessing healthcare information and services.^1^,^4^ A further issue is that people with disabilities may need to rely on family members or carers to access healthcare and keep healthy, and so lack of social support may compound the challenges they face. Consequently, extensive evidence shows that people with disabilities are more likely to experience a range of health conditions, including HIV, childhood diseases, diabetes and cancer.^3^,^5^ As a result of these pathways, emerging findings also show that they have higher mortality rates and lower life expectancy.^6 7^ However, data are still lacking, particularly in sub-Saharan Africa. For instance, a recent systematic review of disability and mortality identified just 31 studies globally, including only three from Africa.^6^ Nevertheless, the review showed that people with disabilities have 2.24-fold (95% confidence interval (CI) 1.84 to 2.72) higher all-cause mortality rates than people without disabilities.

The higher risk of poor health and premature mortality among people with disabilities is important for global health policies and programmes. There are at least 1.3 billion people worldwide with disabilities, making up 16% of the global population.^4^ If this large group continues to be left behind, it will be difficult or impossible to achieve global health targets, such as Universal Health Coverage, Sustainable Development Goal 3 to ‘*ensure healthy lives and promote well-being for all at all ages*’ or programme-specific targets such as those related to HIV control.^3 4 8^ It also matters enormously for people with disabilities and their families if they have poor health and early mortality, especially since the inequities are often large. For example, modelling estimates from the systematic review indicate that the mortality gap translated into a median 13.8 year shorter life expectancy for people with disabilities, which may be even greater in sub-Saharan African settings.^6^ These figures also highlight the infringement of the right to equitable healthcare access as set out in the United Nations Convention on the Rights of Persons with Disabilities^9^ and the laws of most countries.

Certain countries are beginning to make commitments which promote the inclusion of people with disabilities in the health system. In Uganda, for instance, the 2020 Persons with Disabilities Act mandates non-discrimination in provision of health services, accessibility of health units to people with disabilities and the provision of rehabilitation services.^10^ However, these commitments have not yet translated into good healthcare provision for people with disabilities in Uganda.^11^ For example, evidence shows that people with disabilities in Uganda face difficulties accessing sexual and reproductive health services^12^ and HIV care services.^13^ There remains a lack of evidence on inequalities in health facing people with disabilities in Uganda, in particular whether they experience higher mortality rates, which may be indicative of their neglect and exclusion by the health system. Consequently, the aim of this study is to explore the association of disability and mortality, using longitudinal data from the Wellbeing of Older People Study (WOPS) in Uganda.

## Methods

### Study design

WOPS was run by the Medical Research Council/Uganda Virus Research Institute and London School of Hygiene & Tropical Medicine (MRC/UVRI and LSHTM) Uganda Research Unit, as part of the WHO Study on global AGEing and adult health (SAGE). The detailed methodology of WOPS is described elsewhere.^14 15^

In brief, WOPS was an open longitudinal cohort of adults aged at least 50 years old which was established in 2009 in central and south-western Uganda, aiming to research the impact of HIV on health and well-being. Participants were re-interviewed over five waves of data collection until 2022. At baseline, participants were selected randomly for enrolment from two pre-existing cohorts: (1) the General Population Cohort (GPC) located in the predominantly rural Kalungu district (carved out from Masaka district in 2010) in south-western Uganda; (2) the Entebbe cohort located in Wakiso district, a periurban setting in Central Uganda. Additional participants were recruited from older people living with HIV attending The AIDS Support Organization (TASO) in Makasa district and in Wakiso district.

### Participants

To answer the original aim, WOPS consisted of HIV-positive and HIV-negative people aged 50 years and older divided into five groups: (1) have an adult child who died of AIDS; (2) have an adult child who is living with HIV and on antiretroviral therapy (ART); (3) have no child with HIV and are not living with HIV themselves (comparison group); (4) are living with HIV and on ART for at least 1 year and (5) are living with HIV and not on ART (as recruitment occurred before the test-and-treat era). Each group comprised about 100 respondents randomly selected from the study lists at baseline—with half of the respondents recruited from the rural site and half from the urban site. Refusals were less than 1%.

### Data collection and preparation

The five waves of data collection were conducted in the following periods: wave 1 (January 2009–April 2010); wave 2 (February 2013–September 2013); wave 3 (March 2016–November 2016); wave 4 (July 2019–February 2020) and wave 5 (March 2021–February 2022). Data were collected using interviewer-administered questionnaires adapted from the WHO SAGE including sociodemographic and health details and anthropometric measurement (eg, height, weight and waist and hip circumference, blood pressure). Participants were seen at home by interviewers trained in working with older people. If a participant was not found at home during follow-up, interviewers would return another time if other household members or neighbours confirmed the participant was still living at that address. For participants reported to have died, the date of death was noted and a verbal autopsy performed with informed consent from the next of kin. Participants who died or were lost to follow-up were replaced in subsequent waves by new participants matched for age, sex, HIV status and setting.

Data were checked for completeness, consistency and missingness. Cleaning procedures used existing data from the cohorts linked by participant identification numbers to update missing information on deaths, sociodemographic and other characteristics. Related questions were reviewed to verify the most likely values. Overall, less than 5% of data required cleaning, and 12 of the 938 participants ever enrolled in WOPS were assumed to have data missing at random.

### Exposure of interest: disability

At each wave, disability was assessed using the WHO Disability Assessment Schedule instrument (WHO-DAS 2.0), allowing for the measures to vary with time. The WHO-DAS 2.0 12-item score is a standard tool^16^ asking about functional difficulties in the last 30 days in six domains of: mobility, cognition, self-care, interacting with others, household responsibilities and participating in community activities. For each of the 12 questions, responses are on a five-point Likert scale of 0–4: no difficulty, mild, moderate, severe and extreme difficulty/cannot do. The overall functioning for each individual was calculated by summing up their responses and standardising on their expected total responses transformed into a percentage scale. Based on the International Classification of Functioning, Disability and Health (ICF), participants were classified as follows: (1) no disability (<5% impairment) or mild (5%–24%); (2) moderate: 25%–49%; (3) severe to complete disability: >50%. Additionally, respondents reported on whether they experienced functional difficulties using the Washington Group Short Set on Functioning - Enhanced,^17^ which includes the functional domains of: seeing, hearing, walking or climbing stairs, remembering or concentrating, self-care, communication (expressive and receptive), upper body strength and affect (depression and anxiety), with each domain scored as: ‘no difficulty’, ‘some difficulty’, ‘a lot of difficulty’ or ‘cannot do at all’.

### Outcome: mortality

At each follow-up wave, study mobilisers visited the participants’ households to ascertain the vital status of participants, including whether the participant had died since the last wave. A standard WHO verbal autopsy questionnaire was administered by trained interviewers to a close relative or caretaker of the deceased participant. Initially, the study used the WHO-2012 Verbal Autopsy tool but transitioned to the WHO-2016 instrument for wave 4 and wave 5.^18^ The data were electronically captured, then processed to derive the most likely probable cause of death for each case using the InterVA-5 model.^19^ The cause of death was broadly categorised into communicable disease (non-HIV-related), HIV-related, non-communicable diseases (NCDs) and indeterminate.

### Other variables

Data were also collected on participants’ characteristics including sociodemographic, socioeconomic, household, social support, health risk factors and healthcare access. Sociodemographic characteristics included age, sex, marital status, education level achieved, religion and location of participant (rural or urban). The socioeconomic characteristics included current occupation and socioeconomic position (SEP). SEP was operationalised using principal component analysis of housing materials, household ownership of durable assets (eg, bicycle, radio) and ownership of livestock, to create one overall score which was then categorised into tertiles (low/medium/high). Social support characteristics included household size and social index. The social index measure was derived by summing and standardising the total obtained from responses related to participation in social groups, frequency of attendance at religious meetings and services, visiting and being visited and social number of relatives and friends the respondent felt close to. The social index was categorised into tertiles: low, moderate, high. Similarly, the healthcare access index was created by summing and standardising responses to six questions about the participant’s experiences in accessing care including actually getting care, waiting time, degree to which health professionals take time to listen and to explain about the condition and its treatment and their degree of satisfaction with services. The healthcare access index was categorised into tertiles: ‘good access’, ‘moderate access’ and ‘poor access’.

In measuring systolic and diastolic blood pressures, three measurements of each were undertaken and the mean of the two closest values was assigned to the participant. Participants were classified as having hypertension if they had a systolic blood pressure ≥140 mm Hg, a diastolic blood pressure ≥90 mm Hg or reported taking antihypertensive medication. Body mass index was categorised into: <18.5 kg/m^2^ (underweight), 18.5–24.9 kg/m^2^ (healthy weight), 25–29.9 kg/m^2^ (overweight) and ≥30 kg/m^2^ (obese). Angina and stroke were defined as either an existing clinical diagnosis or a suggestive history at interview (chest pain on exertion for angina; episode of hemiparesis or hemisensory loss lasting at least 24 hours for stroke) and a composite variable was created indicating with or without cardiovascular disease. Participants also self-reported whether they had ever been diagnosed and were on treatment for diabetes and chronic lung disease. Chronic lung disease included emphysema, bronchitis or chronic obstructive pulmonary disease. HIV testing was conducted at all waves following the Uganda Ministry of Health testing algorithm for HIV rapid testing.^14^

### Statistical analyses

In this cohort, participants contributed exposure time from the date of enrolment, where their first assessment for disability was done, to the date of their last interview or death. Participants lost to follow-up with no subsequent follow-up visit were censored at a date 6 months after their last interview date to allow them to contribute exposure time. Inspection of the hazard function for mortality in this population showed an exponential increase in hazard ratios (HRs) with age. Consequently, the multivariable Gompertz regression model was fitted to estimate the association between mortality (dependent variable) and disability (independent variable).^20^ Models were sequentially adjusted for variables considered to be relevant to the association between disability and mortality. These variables were included in the model based on a hypothesised pathway, starting with the more distal to more proximate factors in the association. Factors were retained in the final model if they showed a 15% change in the coefficient of disability, suggesting potential confounding. We also assessed for effect modification by sex and HIV status. Cause-specific mortality rates were obtained and models fitted based on a competing risk analysis to account for the alternative causes of death. To assess the potential relationships between covariates and disability, we considered the most severe disability for each participant over the observation period, given disability was time-varying. Multinomial logistical regression models were fitted to obtain the age-sex adjusted p values.

### Patient and public involvement

A community advisory board was established for the study, which included older persons representing different sections of the target population. This group provided input on study conduct, reporting and dissemination of the research.

## Results

### Participants’ enrolment and follow-up

Across the five waves, 938 WOPS study participants were cumulatively enrolled and included in this analysis (online supplemental figure 1). By design, the majority were enrolled in wave 1 (54%, n=510), and the median follow-up time for all participants was 8.0 years (interquartile range (IQR): 3.2–11.5; maximum 12.8 years).

Participant characteristics are summarised in table 1. 59% were female. The median age of participants was 62 years (IQR: 55–72), and this did not differ by sex. Participants most frequently engaged in farming occupations (65%), the majority reported actively working full-time (68%), and most had not attended education beyond the primary level (78%). A large proportion were living in households with six or more members (37%). A quarter of the participants reported having been diagnosed with more than one chronic health condition including HIV, while another quarter reported none.

**Table 1:** Sociodemographic and economic characteristics of WOPS participants by highest disability severity during follow-up

Characteristic	All participants	Severity of disability			Overall difference in disability outcomes P value
	n (%)	None/mild (<25% impairment)	Moderate (25–49% impairment)	Severe (≥50% impairment)	
		n (%)	n (%), P value	n (%), P value	
Number of respondents (row %)	**938**	**287 (31%)**	**350 (37%)**	**301 (32%)**	
Demographic					
Sex			**p<0.001**	**p<0.001**	**p<0.001**
Men	384 (41%)	172 (60%)	117 (33%)	95 (32%)	
Women	554 (59%)	115 (40%)	233 (67%)	206 (68%)	
Age (in years)			**p<0.001**	**p<0.001**	**p<0.001**
50–59	357 (38%)	156 (54%)	129 (37%)	72 (24%)	
60–69	283 (30%)	86 (30%)	132 (38%)	65 (22%)	
70+	298 (32%)	45 (16%)	89 (25%)	164 (55%)	
Marital status			**p=0.87**	**p=0.10**	**p=0.21**
Married/cohabiting	342 (37%)	143 (50%)	121 (35%)	78 (26%)	
Divorced/separated	199 (21%)	59 (21%)	79 (23%)	61 (20%)	
Widowed/never married	397 (42%)	85 (30%)	150 (43%)	162 (54%)	
Education level achieved			**p=0.42**	**p=0.04**	**p=0.05**
None	192 (21%)	43 (15%)	63 (18%)	86 (29%)	
Primary	538 (57%)	157 (55%)	209 (60%)	172 (57%)	
Secondary or higher	208 (22%)	87 (30%)	78 (22%)	43 (14%)	
Religion			**p=0.37**	**p=0.18**	**p=0.36**
Catholic	560 (60%)	167 (58%)	205 (59%)	188 (63%)	
Protestant	221 (24%)	67 (23%)	82 (23%)	72 (24%)	
Islam	101 (11%)	30 (11%)	43 (12%)	28 (9%)	
Other	56 (6%)	23 (8%)	20 (6%)	13 (4%)	
Geographical setting			**p=0.87**	**p=0.12**	**p=0.33**
Kyamulibwa (rural)	433 (46%)	125 (44%)	156 (45%)	152 (51%)	
Entebbe (urban)	374 (40%)	107 (37%)	141 (40%)	126 (42%)	
Masaka (urban)	131 (14%)	55 (19%)	53 (15%)	23 (8%)	
Economic					
Current occupation			**p=0.84**	**p=0.30**	**p=0.67**
Farming only	358 (38%)	110 (38%)	130 (37%)	118 (39%)	
Farming and other	251 (27%)	84 (29%)	99 (28%)	68 (23%)	
Other, non-farming	220 (24%)	70 (24%)	84 (24%)	66 (22%)	
None	109 (12%)	23 (8%)	37 (11%)	49 (16%)	
Whether still working*			**p=0.10**	**p=0.001**	**p=0.006**
Working full-time	630 (68%)	219 (77%)	242 (70%)	169 (57%)	
Working part-time	143 (16%)	20 (7%)	48 (14%)	75 (25%)	
Self-employed	69 (8%)	30 (11%)	26 (8%)	13 (4%)	
Not working	82 (9%)	14 (5%)	30 (9%)	38 (13%)	
Household Wealth Index			**p=0.44**	**p=0.03**	**p=0.04**
Lowest tertile	310 (33%)	81 (28%)	111 (32%)	118 (39%)	
Middle tertile	318 (34%)	110 (38%)	115 (33%)	93 (31%)	
Highest tertile	310 (33%)	96 (33%)	124 (35%)	90 (30%)	
Social					
Household size			**p=0.02**	**p=0.11**	**p=0.08**
1	118 (13%)	39 (14%)	38 (11%)	41 (14%)	
2–5	470 (50%)	158 (55%)	165 (47%)	147 (49%)	
6+	350 (37%)	90 (31%)	147 (42%)	113 (38%)	
Participation in any social groups			**p=0.18**	**p=0.004**	**p=0.02**
Yes	459 (49%)	162 (56%)	178 (51%)	119 (40%)	
No	479 (51%)	125 (44%)	172 (49%)	182 (61%)	
Frequency of attending religious meetings			**p=0.12**	**p=0.02**	**p=0.01**
Never/almost	479 (51%)	125 (44%)	172 (49%)	182 (61%)	
Once or twice a year	58 (6%)	21 (7%)	15 (4%)	22 (7%)	
Every few months	61 (7%)	28 (10%)	20 (6%)	13 (4%)	
Once or twice a month	340 (36%)	113 (39%)	143 (41%)	84 (28%)	
Washington Group Short Set on Functioning - Enhanced disability indicator			**p<0.001**	**p<0.001**	**p<0.001**
Without disability	331 (35%)	191 (67%)	100 (29%)	40 (13%)	
With disability	607 (65%)	96 (33%)	250 (71%)	261 (87%)	

* Data on this variable were missing for 14 participants.

WOPS, Wellbeing of Older People Study.

### Disability levels, domains and associated factors

Overall, 32% of the participants were classified with severe disability, while 37% with moderate disability and 31% with none/mild disability. Compared with those with none/mild disability, participants with severe disability at any point during follow-up were more likely to be female, older, less educated or unemployed and of lower socioeconomic status (table 1; online supplemental figure 2). They were also less likely to participate in social groups or attend religious meetings regularly. There was a high level of overlap between reported disability using WHO-DAS and the Washington Group Short Set on Functioning-Enhanced. Virtually all the participants across the five waves classified as having severe disability using WHO-DAS were also categorised as having disability using the Washington Group Short Set on Functioning-Enhanced (98%, n=439). The most commonly reported functional difficulties among people with severe disability were upper-body strength (74%), vision (58%) and mobility (58%) (online supplemental figure 3). Furthermore, 37% of participants reported severe or extreme difficulties in two or more domains encompassing vision, cognition, movement, self-care, upper-body strength, depression and anxiety.

Table 2 shows baseline health status, health risk factors and health access by severity of disability. There was little difference in self-reported health conditions between people with disabilities (moderate or severe) and those with none/mild disability. However, people with disabilities reported higher prevalences of arthritis and lower prevalences of HIV compared with those with none/mild disability. Participants living with HIV were notably younger (<60 years), which may explain their lower likelihood of experiencing disabilities compared with those who were older and also more likely to be HIV negative.

**Table 2:** Health status at enrolment by highest disability severity during follow-up

Characteristic	All participants	Severity of disability			Overall difference in disability outcomes P value*
		None/mild (<25% impairment)	Moderate (25–49% impairment)	Severe (≥50% impairment)	
	n (%)	n (%)	n (%), P value*	n (%), P value*	
Number of respondents (row %)	**938**	**287** (**31%**)	**350** (**37%**)	**301** (**32%**)	
Self-reported health					
Number of health conditions†			**p=0.72**	**p=0.83**	**p=0.84**
None	250 (27%)	63 (22%)	86 (25%)	101 (34%)	
One	468 (50%	158 (55%)	172 (49%)	138 (46%)	
More than one	220 (24%)	66 (23%)	92 (26%)	62 (21%)	
HIV status			**p=0.34**	**p=0.006**	**p=0.02**
HIV negative	455 (49%)	98 (34%)	161 (46%)	196 (65%)	
HIV positive	483 (52%)	189 (66%)	189 (54%)	105 (35%)	
Arthritis			**p=0.004**	**p=0.01**	**p=0.01**
No	895 (95%)	284 (99%)	327 (93%)	284 (94%)	
Yes	43 (5%)	3 (1%)	23 (7%)	17 (6%)	
Chronic lung disease			**p=0.77**	**p=0.92**	**p=0.92**
No	888 (95%)	268 (93%)	333 (95%)	287 (95%)	
Yes	50 (5%)	19 (7%)	17 (5%)	14 (5%)	
Stroke			**p=1.00**	**p=0.22**	**p=0.29**
No	920 (98%)	283 (99%)	345 (99%)	292 (97%)	
Yes	18 (2%)	4 (1%)	5 (1%)	9 (3%)	
Angina			**p=0.24**	**p=0.14**	**p=0.34**
No	921 (98%)	285 (99%)	343 (98%)	293 (97%)	
Yes	17 (2%)	2 (1%)	7 (2%)	8 (3%)	
Diabetes			**p=0.53**	**p=0.44**	**p=0.29**
No	891 (95%)	275 (96%)	336 (96%)	280 (93%)	
Yes	47 (5%)	12 (4%)	14 (4%)	21 (7%)	
Hypertension			**p=0.40**	**p=0.93**	**p=0.54**
No	650 (69%)	216 (75%)	233 (67%)	201 (67%)	
Yes	288 (31%)	71 (25%)	117 (33%)	100 (33%)	
Clinical examination					
Hypertension level based on systolic and diastolic pressure			**p=0.19**	**p=0.47**	**p=0.32**
Normal	504 (54%)	168 (59%)	182 (52%)	154 (51%)	
Mild	314 (34%)	87 (30%)	128 (37%)	99 (33%)	
Grade 2/3 hypertension	120 (13%)	32 (11%)	40 (11%)	48 (16%)	
Body Mass Index (kg/m^2^)			**p=0.33**	**p=0.008**	**p=0.02**
Underweight	130 (15%)	37 (14%)	42 (13%)	51 (19%)	
Normal	522 (61%)	180 (69%)	201 (62%)	141 (52%)	
Overweight	209 (24%)	45 (17%)	84 (26%)	80 (29%)	
Health risk factors					
Tobacco use			**p=0.03**	**p=0.03**	**p=0.009**
Never smoked	654 (70%)	195 (68%)	253 (72%)	206 (68%)	
Stopped smoking	140 (15%)	58 (20%)	37 (11%)	45 (15%)	
Currently smokes	144 (15%)	34 (12%)	60 (17%)	50 (17%)	
Alcohol consumption			**p=0.45**	**p=0.25**	**p=0.52**
Never drank alcohol	265 (28%)	75 (26%)	102 (29%)	88 (29%)	
Less than once a month	448 (48%)	126 (44%)	174 (50%)	148 (49%)	
At least once a month	225 (24%)	86 (30%)	74 (21%)	65 (22%)	
Hunger in the last 12 months			**p=0.08**	**p=0.003**	**p=0.01**
Never lacked food	705 (75%)	231 (81%)	264 (75%)	210 (70%)	
Only some months	128 (14%)	29 (10%)	52 (15%)	47 (16%)	
Almost every month	105 (11%)	27 (9%)	34 (10%)	44 (15%)	
Healthcare Access Index			**p=0.001**	**p<0.001**	**p<0.001**
Poor access	356 (38%)	76 (27%)	142 (40%)	138 (46%)	
Moderate access	402 (43%)	135 (47%)	153 (44%)	114 (38%)	
Good access	180 (19%)	76 (27%)	55 (16%)	49 (16%)	

* P values are age and sex adjusted from a multinomial regression model.

† Health conditions measured: HIV, arthritis, chronic lung disease, stroke, angina, diabetes, hypertension.

Clinical examination of participants revealed no statistically significant difference in hypertension by disability status, but there was a higher prevalence of being underweight and overweight among people with severe disability compared with those with none/mild disability. In addition, people with disabilities reported slightly higher prevalences of current tobacco use and hunger during some months or almost every month in the last 12 months. Reported healthcare access was substantially worse among people with moderate (40%) or severe disability (46%) compared with those with no/mild disability (27%).

### All-cause mortality

The 12-year follow-up period accumulated 6414 person-years of observation in which 153 deaths occurred (table 3). The overall mortality rate was 2.4 deaths per 100 person-years (95% CI 2.0 to 2.8). Age-adjusted and sex-adjusted mortality rates by sociodemographic, economic, social and health characteristics are shown in online supplemental table 1. The rates were similar for participants classified with none/mild disability (1.3 deaths/100 person-years) and those with moderate disability (1.7 deaths/100 person-years), but substantially higher among those with severe disability (7.5 deaths/100 person-years) even after adjusting for age and sex. When disability was disaggregated into broad functional domains, vision and physical impairments remained independently associated with higher mortality after adjustment for vision, physical and psychological difficulties, age, sex and location (online supplemental table 2). However, the effect of psychological difficulties was attenuated. In addition, participants with persistently severe disability showed excess mortality risk (10.1/100 person-years) compared with those with persistently non-severe disability (1.6/100 person-years), improved disability (0.8/100 person-years) and worsened state of disability (2.6/100 person-years; p<0.001; online supplemental table 3).

**Table 3:** The relative risk of all-cause mortality by WHO Disability Assessment Schedule classification (WHODAS) among adults aged 50 years and above

WHODAS classification	Number of deaths/person-years of follow-up	Mortality rate per 100 person-years (95% CI)	Age and sex adjusted HR (95% CI)	+Education, marital status, religion, residence, household wealth index, current occupation HR (95% CI)	+Social support index HR (95% CI)	+Healthcare care access index HR (95% CI)	+Health risk factors (tobacco, alcohol, BMI) HR (95% CI)	+Poor health conditions (fully adjusted model)* HR (95% CI)
OVERALL	153/6414	2.4 (2.0 to 2.8)						
None/mild: <25% impairment	43/3300	1.3 (1.0 to 1.8)	Reference	Reference	Reference	Reference	Reference	Reference
Moderate: 25%–49% impairment	35/2115	1.7 (1.2 to 2.3)	1.16 (0.73 to 1.83)	1.06 (0.66 to 1.69)	1.04 (0.65 to 1.67)	1.02 (0.64 to 1.64)	1.04 (0.64 to 1.68)	1.08 (0.68 to 1.75)
Severe: >50% impairment	75/998	7.5 (6.0 to 9.4)	3.88 (2.50 to 6.02)	3.32 (2.09 to 5.3)	2.99 (1.86 to 4.79)	2.93 (1.82 to 4.71)	2.97 (1.82 to 4.83)	3.08 (1.92 to 4.93)
Likelihood ratio test p value			p<0.001	p<0.001	p<0.001	p<0.001	p<0.001	p<0.001†

* Adjusted for sex, age, marital status, education level achieved, location (rural, urban), current occupation, Social Support Index, health conditions, Healthcare Access Index, tobacco use, alcohol consumption and HIV status.

† P value for trend test=0.009.

BMI, body mass index; HR, hazard ratio.

Participants with severe disability showed substantially worse survival probabilities across age compared with those with either moderate or none/mild disability regardless of participants’ sex, as shown by the survival curves (Logrank test p value <0.001; online supplemental figure 4). In both females and males, the median survival age was considerably lower among participants with severe disability (females: 73.6 years; males: 53.0) than among those with moderate disability (females: 93.3 years; males: 86.6) or none/mild disability (females: 87.7 years; males: 85.3; red dotted lines in online supplemental figure 4a,b). This appears to indicate a survival disadvantage in participants with severe disability compared with those with either none/mild disability or moderate disability. These observations also suggest that men with severe disability were more likely to die earlier than women with severe disability. However, there was no evidence to suggest a difference in the survival probabilities for people with moderate disability and those with none/mild disability (p=0.90).

The effect of severe disability on mortality and the associated strength of evidence remained consistently strong as models were adjusted for additional variables (table 3). The age-sex-adjusted analyses suggested a 3.88-fold higher risk of death at any age (HR 3.88; 95% CI 2.50 to 6.02; p value <0.001) among people with severe than none/mild disability. Adjustment for sociodemographic, economic, social support and health status/access/risk factors attenuated the association, but did not explain it in full (HR for fully adjusted model 3.08, 95% CI 1.92 to 4.93; p value <0.001).

To assess whether the COVID-19 period influenced mortality patterns, we compared mortality before and during the pandemic. Among these participants aged ≥50 years, the mortality rate was higher during the COVID-19 period (2.9 deaths per 100 person-years, 1 April 2020–28 February 2022) compared with the pre-COVID-19 period (1.7 deaths per 100 person-years, 28 November 2017–31 March 2020). In the multivariable model, there was no statistically significant evidence to suggest that the association between disability and mortality differed before and during the pandemic (interaction p=0.112).

### Cause-specific mortality

Verbal autopsy analysis showed that the majority of the 153 deaths were due to NCDs (53%, n=81), followed by communicable diseases (not HIV–23%, n=35), HIV (12%, n=19) or indeterminate causes (12%, n=18) (table 4; online supplemental figure 5). The observed distribution of causes of death might not reflect the actual distribution in the general population, even among older people, given the oversampling of HIV patients in the current study. Across all the broad categories for causes of death, people with severe disability consistently had an elevated mortality rate compared with those with either moderate or none/mild disability (table 4). Among people with severe disability, mortality rates due to NCDs (3.8/100 person-years; 95% CI 2.8 to 5.2) were at least two times higher than rates due to non-HIV communicable diseases (1.6/100 person-years; 95% CI 1.0 to 2.6) or HIV/AIDS (1.0/100 person-years; 95% CI 0.5 to 1.9). While the effect of severe disability (compared with none/mild disability) on HIV-related cause of death (fully adjusted model, adjusted HR (aHR) 8.96, 95% CI 2.52 to 31.83) and indeterminate causes of death (aHR 6.89, 95% CI 1.63 to 29.1) were more apparent, the wide CIs indicate less precise estimates and should be interpreted with caution. The risk of mortality due to both NCDs (aHR 2.29, 95% CI 1.20 to 4.37) and communicable diseases excluding HIV (aHR 2.24, 95% CI 0.80 to 6.25) was twice in those with severe disability compared with those classified with none/mild disability. However, the weak evidence of association with mortality due to communicable diseases was likely because of the small number of deaths among those with none/mild disability. Across all broad causes of death, there was no evidence suggesting differences in mortality between people with moderate disabilities and those with none/mild disability.

**Table 4:** Adjusted relative risk of cause-specific mortality by the WHO Disability Assessment Schedule classification (WHODAS) among older people aged 50 years and above

By cause of death: adjusted for age and sex													
Cause of death		Communicable disease (excluding HIV)			HIV			Non-communicable disease			Indeterminant		
Disability severity based on WHODAS 2.0	No. deaths/person-years of follow-up	n	Rate per 100 person-years (95% CI)	aHR (95% CI)	n	Rate per 100 person-years (95% CI)	aHR (95% CI)	n	Rate per 100 person-years (95% CI)	aHR (95% CI)	n	Rate per 100 person-years (95% CI)	aHR (95% CI)
All participants	153/6414	35	0.6 (0.4 to 0.8)		19	0.3 (0.2 to 0.5)		81	1.3 (1.0 to 1.6)		18	0.3 (0.2 to 0.4)	
None/mild: <25% impairment	43/3300	9	0.3 (0.1 to 0.5)	Reference	5	0.2 (0.1 to 0.4)	Reference	24	0.7 (0.5 to 1.1)	Reference	5	0.2 (0.1 to 0.4)	Reference
Moderate: 25%–49% impairment	35/2115	10	0.5 (0.3 to 0.9)	1.22 (0.46 to 3.22)	4	0.2 (0.1 to 0.5)	1.63 (0.41 to 6.49)	19	0.9 (0.6 to 1.4)	0.93 (0.49 to 1.77)	2	0.1 (0.02 to 0.4)	0.81 (0.14 to 4.67)
Severe: >50% impairment	75/998	16	1.6 (1.0 to 2.6)	2.24 (0.80 to 6.25)	10	1.0 (0.5 to 1.9)	8.96 (2.52 to 31.83)	38	3.8 (2.8 to 5.2)	2.29 (1.20 to 4.37)	11	1.1 (0.6 to 2.0)	6.89 (1.63 to 29.1)
Likelihood ratio test p value				**0.24**			**0.001**			**0.006**			**0.007**

Adjusted for sex, age, marital status, education level achieved, location(rural, urban), current occupation, Social Support Index, Healthcare Access Index, tobacco use, alcohol consumption and HIV status. The main contributing diseases under communicable diseases (excluding HIV) were respiratory diseases and more so among participants with severe disability (62.5%). In the non-communicable disease cause of death, the main contributing diseases were cardiovascular diseases (50.0%).

## Discussion

Our findings show strong evidence for an association between severe disability and risk of death. People with severe disability were associated with an almost four times higher risk of all-cause mortality (age-sex aHR 3.88; 95% CI 2.50 to 6.02; p value <0.001) than those with mild or no disability, which attenuated but did not disappear after adjusting for socioeconomic factors, health access, health risk factors and prior conditions (HR for fully adjusted model 3.08, 95% CI 1.92 to 4.93; p value <0.001). The weakening of the association suggests that the identified pathways may help to explain the relationship of disability and mortality—including underlying health conditions, poverty, lack of social support and barriers to accessing healthcare services. The persistent association in the fully adjusted models may indicate either the presence of residual confounding or additional unmeasured explanatory factors (eg, coverage of healthcare services). Notably, there was no evidence to suggest that people with moderate disability had an increased risk of death than those with mild or no disability. In this study population, NCDs were the main cause of death when compared with causes such as HIV, other communicable diseases and indeterminate causes. The study findings also showed that participants with disabilities were associated with worse socioeconomic conditions, lower educational attainment, higher levels of poverty, weaker social networks, greater barriers to accessing healthcare and had a higher prevalence of risk factors for ill-health (eg, being under or overweight, tobacco use and hunger), all of which are known risk factors for morbidity and mortality.

In general, scientific evidence is limited on the relationship between disability status and mortality, particularly for sub-Saharan Africa. The systematic review by Kuper *et al* (2024) only found three papers out of 31 that examined the association between disability and all-cause mortality in the WHO Africa Region and none from Uganda.^6^ Pooled global estimates from the meta-analysis were lower than in our study, with all-cause mortality found to be 2.24 times higher (95% CI 1.84 to 2.72) among people with disabilities in comparison to those without disabilities.^6^ The systematic review identified 11 papers on cause-specific mortality, none of which were from Africa and only one was from a low- or middle-income country setting. Cause-specific mortality in the review remained higher for people with disabilities for a range of diseases including cancer, cardiovascular disease, suicide and COVID-19.^6^ This is consistent with findings from our study, whereby mortality is higher among people with disabilities across the different causes of death, including HIV, other communicable diseases, NCDs and indeterminate causes. These findings suggest that the link between disability status and mortality is not disease-specific.

Despite a greater risk of mortality among people with severe disability, there was no evidence in this study that people with severe disability were more likely to have poorer health. This could be attributed to the older age of the sample, where ill health and having multiple health conditions are likely to be common,^21^ making it harder to distinguish health differences by disability status. The lack of evidence for worse health outcomes among people with disabilities in this study contrasts with previous findings, which typically show poorer health among those with disabilities compared with those without.^2^ Our research may be more reflective of the older population in our sample, in contrast to the wider literature which focuses mostly on adult populations. This is important as barriers to accessing health among older people in Uganda are similar to the barriers experienced by people with disabilities, including the distance to the health facility, the attitudes of healthcare workers and the lack of specialised care, and so differences in health outcomes may be attenuated in an older group.^22^,^25^

Our study also revealed that people with disabilities in this cohort had a lower prevalence of HIV infection than their non-disabled counterparts, despite higher HIV-related mortality, which is in contrast to the broader literature.^26^ However, since this cohort was designed to over-sample people living with HIV, the lack of association between disability and HIV status in this study may not fully represent the broader population.

The classification of causes of death relied on the WHO verbal autopsy tool, which is widely used in low-resource settings but may be less specific in older adults where deaths often follow gradual decline and multiple comorbidities. In our cohort, 10 of 153 deaths lacked completed verbal autopsy data, and among the remaining 143, eight (5.6%) were classified as indeterminate—slightly higher than expected but comparable to the GPC, where indeterminate causes accounted for around 2–3% of adult deaths. While verbal autopsy remains a valuable method for mortality surveillance, refinements to algorithms for older populations could improve accuracy.

Hypertension and HIV were objectively measured, whereas other chronic conditions such as stroke, angina, diabetes and chronic lung disease were self-reported. This difference in ascertainment may have led to underdiagnosis and attenuated associations with mortality. Future studies incorporating clinical confirmation of NCDs will be important to improve precision in estimating comorbidity and mortality risk among older adults in sub-Saharan Africa.

### Strengths and limitations

This study is one of the first to explore the relationship between disability and mortality in sub-Saharan Africa, contributing to a currently limited body of evidence. The cohort provides a median of 8 years of follow-up and five waves of data collection for participants since the cohort was initially established in 2009 (representing 54% of the sample). Importantly, all participant deaths were verified, with the cause of death determined through verbal autopsy.^19^ The use of WHODAS provides a consistent measure of disability with which to make comparisons across each wave, enabling meaningful comparisons over time. WHODAS uses a continuous disability scale, reflecting real-life variation—although for the purposes of analysis, participants are divided into groups dependent on severity. Although we were able to distinguish broad functional domains of disability, data on the underlying cause, onset or type of impairment (eg, congenital vs acquired) were not collected. This limited our ability to explore whether mortality patterns varied by impairment type or duration and should be considered in future studies.

Each wave of the survey included validated questions on a wide range of sociodemographic, socioeconomic, health and social support characteristics, enabling the investigation of a wide range of variables to elucidate the association between disability status and mortality. Nevertheless, some residual or unexplained confounding is likely, as not all relevant indicators were included or would have fully captured complex constructs such as barriers to healthcare access or social disadvantage. While socioeconomic disadvantage and limited healthcare access may contribute to the elevated mortality risk among people with disabilities, these factors do not appear to fully explain the observed association. Consequently, it was not possible to estimate what proportion of deaths might have been avoidable through improved access to care.

The focus of this longitudinal study in Uganda is on the health and well-being of older people, with comparisons based on HIV status. Consequently, all participants in the cohort are aged 50 years and above, with many participants either living with HIV (52%) or affected by HIV. Therefore, our findings may not be generalisable to those under 50 years of age or for population groups with lower prevalences of HIV.

### Implications for research and practice

Our results show that the risk of mortality is unacceptably higher for older people living with severe disability in Uganda. The Ugandan 2020 Persons with Disabilities Act states that health facilities must be accessible to people with disabilities and the provision of healthcare must be non-discriminatory. Despite this mandate, it is evident that further work is needed to strengthen the health system in Uganda to better include people with disabilities. To close this mortality gap, further research is also needed to understand what works—specifically what proportion of deaths are avoidable through promotive, preventive and curative health services.

## Conclusions

This study reveals a significantly higher all-cause mortality risk among older adults with severe disability in Uganda. While socioeconomic disadvantages and barriers to healthcare contribute to this elevated risk, they do not fully explain the association, highlighting the need for further research into other contributing factors. These findings underscore the need to strengthen the health system in Uganda to better support people with disabilities and reduce the mortality gap, particularly in sub-Saharan Africa where data remain limited.

## Supplementary material

10.1136/bmjgh-2025-019802 online supplemental file 1

10.1136/bmjgh-2025-019802 online supplemental file 2

## Data Availability

Data are available in a public, open access repository.
