# Supplementary material for: Association of disability and 12-year all-cause and cause-specific mortality: analyses from the Wellbeing of Older People cohort study in Uganda
Source: BMJ Glob Health. 2026 Jan 23;11(1):e019802. doi: 10.1136/bmjgh-2025-019802 (PMC12853530; doi:10.1136/bmjgh-2025-019802)
Supplement: online supplemental file 1 [file bmjgh-11-1-s001.docx]

**Supplementary Materials**

**Supplementary Figure 1: Enrolment and follow-up of 938 participants in the WOPS study**


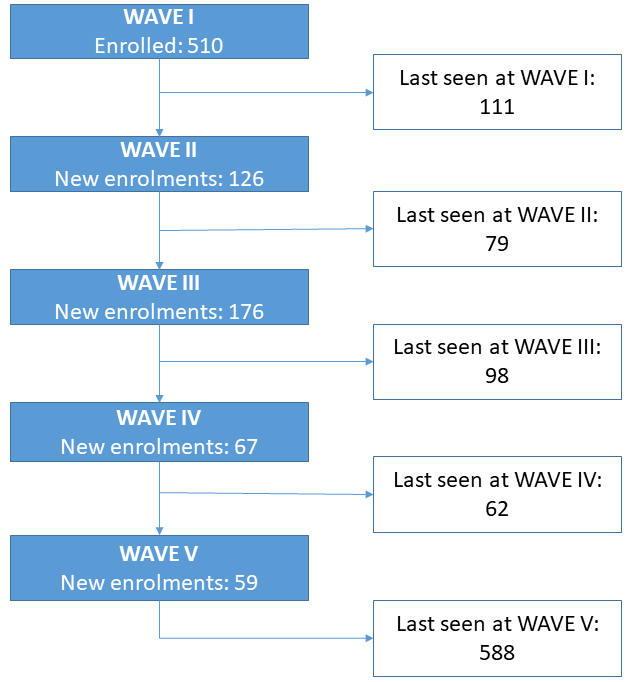


**Supplementary Figure 2: World Health Organization Disability Assessment Schedule (WHODAS) classification by sex, and age**


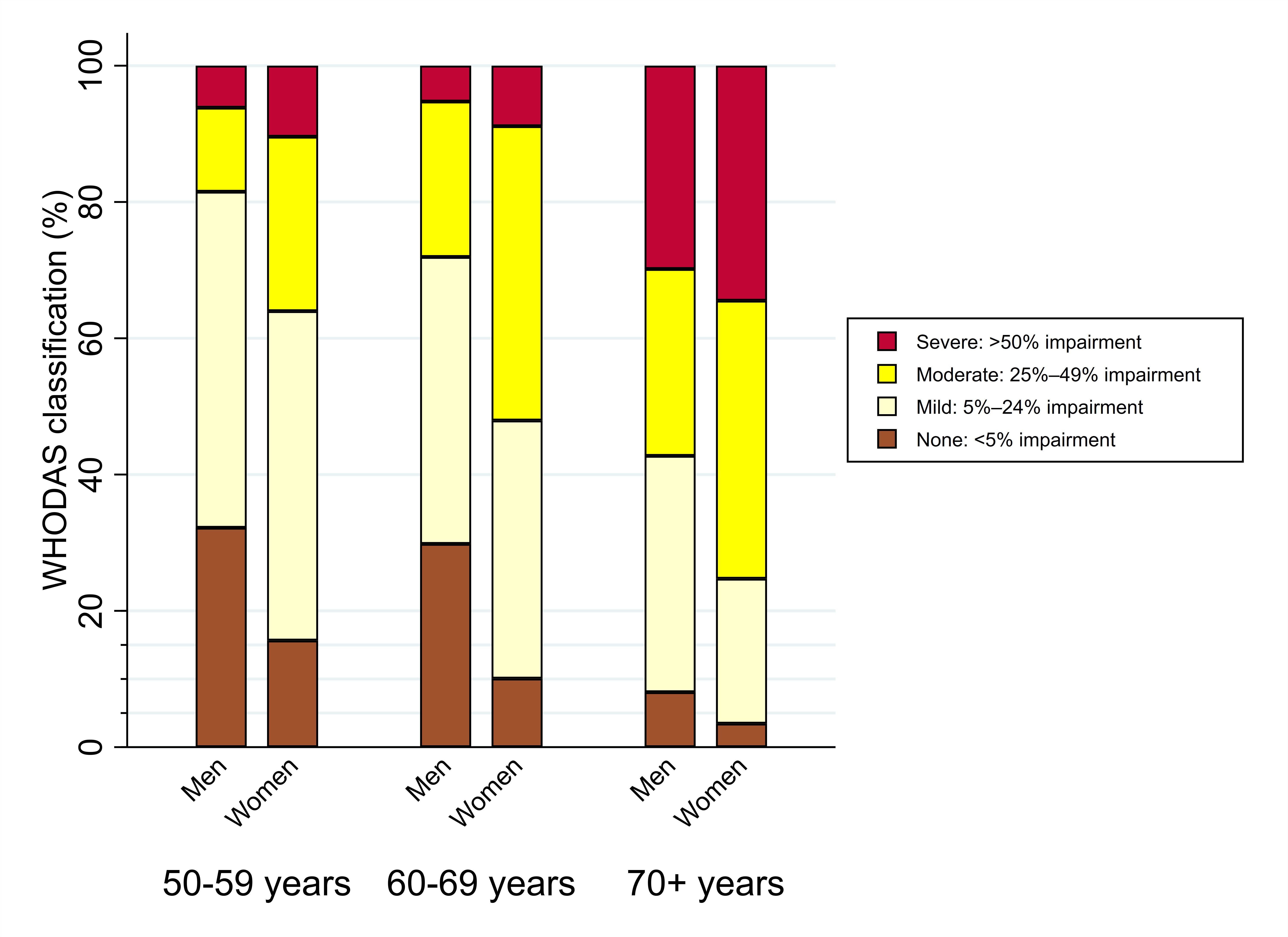


**Supplementary Figure 3: Proportion reporting impairments in each WHODAS classification**


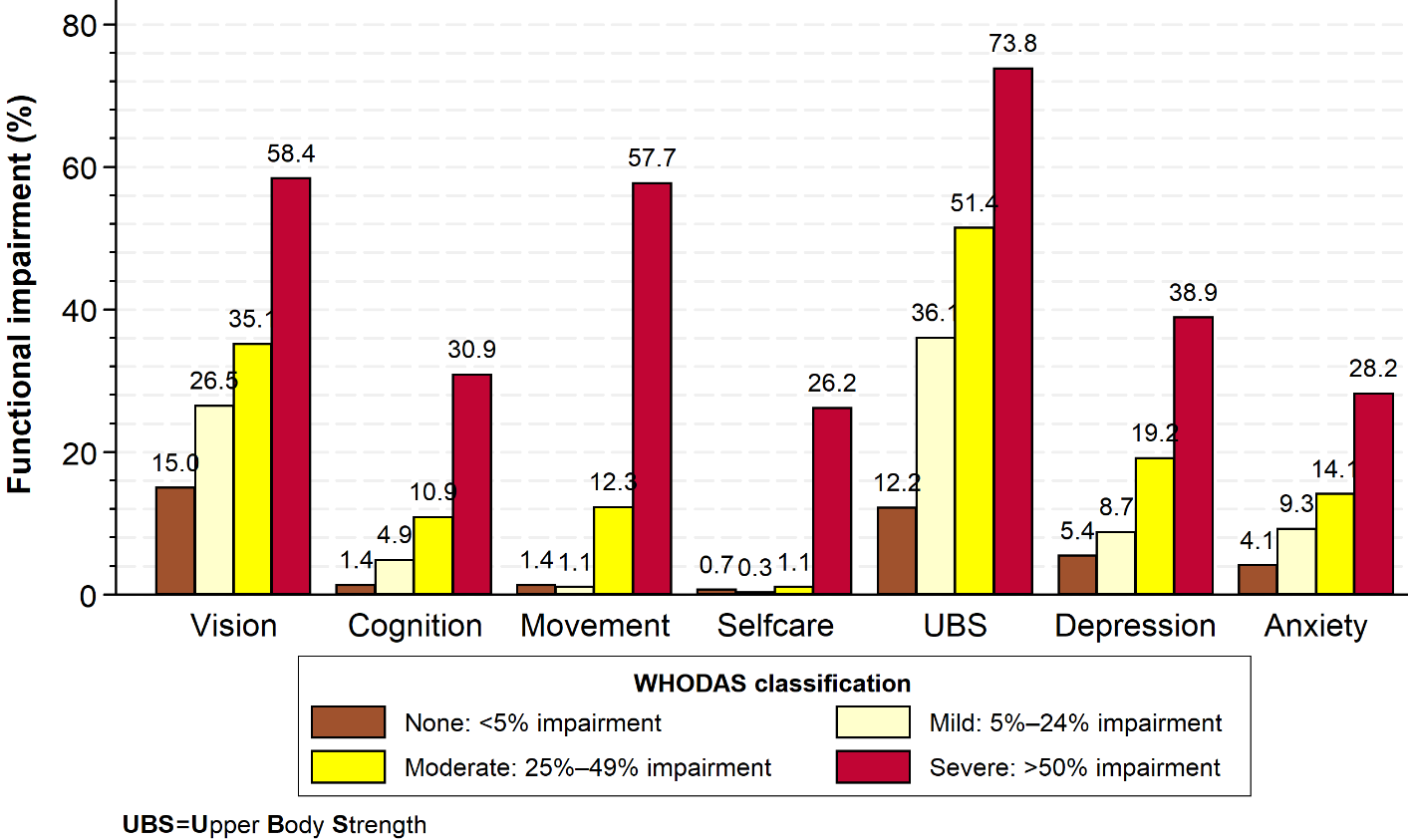


**Supplementary Table 1: Mortality rates (per 100 person-years) and hazard ratios adjusted by age and sex for socio-demographic characteristics among older people aged 50 years and above.**

| **Characteristic** | **Categories** | **Deaths/ Person-years** | **Mortality Rates per 100 person-years**  **(95% CI)** | **Age-sex adjusted Hazard Ratio (95% CI)** | **LR-test p-value** |
| --- | --- | --- | --- | --- | --- |
| Overall |  | 153/6414 | 2.4 (2.0-2.8) |  |  |
|  |  |  |  |  |  |
| **DEMOGRAPHIC** |  |  |  |  |  |
| WHODAS classification of impairment | Mild/none: <25% | 43/3300 | 1.3 (1.0-1.8) | 1 | <0.001 |
|  | Moderate: 25%-49% | 35/2115 | 1.7 (1.2-2.3) | 1.13 (0.71-1.79) |  |
|  | Severe: >50% | 75/998 | 7.5 (6.0-9.4) | 3.90 (2.53-6.01) |  |
|  |  |  |  |  |  |
| Sex | Men | 73/2472 | 3.0 (2.3-3.6) | 1 | 0.03 |
|  | Women | 80/3942 | 2.0 (1.5-2.4) | 0.70 (0.51-0.97) |  |
|  |  |  |  |  |  |
| Age (in years) | 50-59 | 21/2079 | 1.0 (0.7-1.5) | 1 | 0.31 |
|  | 60-69 | 32/2213 | 1.4 (1.0-2.0) | 0.90 (0.50-1.62) |  |
|  | 70+ | 100/2122 | 4.7 (3.9-5.7) | 1.35 (0.65-2.78) |  |
|  |  |  |  |  |  |
| Marital Status | Married/Cohabiting | 45/2200 | 2.0 (1.5-2.7) | 1 | 0.06 |
|  | Divorced/Separated | 25/1423 | 1.8 (1.2-2.6) | 1.17 (0.70-1.95) |  |
|  | Widow/never married | 83/2790 | 2.8 (2.4-3.7) | 1.66 (1.08-2.56) |  |
|  |  |  |  |  |  |
| Religious denomination | Catholic | 100/3813 | 2.6 (2.2-3.2) | 1 | 0.18 |
|  | Protestant | 30/1510 | 2.0 (1.4-2.8) | 0.71 (0.47-1.07) |  |
|  | Islam | 17/640 | 2.7 (1.7-4.3) | 1.05 (0.63-1.77) |  |
|  | Other | 6 /451 | 1.3 (0.6-3.0) | 0.56 (0.25-1.28) |  |
|  |  |  |  |  |  |
| Education level achieved | None | 50/1243 | 4.0 (3.0-5.3) | 1 | 0.009 |
|  | Primary | 78/3713 | 2.1 (1.7-2.6) | 0.58 (0.41-0.84) |  |
|  | Secondary or higher | 25/1458 | 1.7 (1.2-2.5) | 0.54 (0.33-0.88) |  |
|  |  |  |  |  |  |
| Location | Rural | 75/2980 | 2.5 (2.0-3.2) | 1 | 0.59 |
|  | Urban | 78/3433 | 2.3 (1.8-2.8) | 1.09 (0.79-1.50) |  |
|  |  |  |  |  |  |
| **ECONOMIC** |  |  |  |  |  |
| Current occupation | Farming only | 49/2374 | 2.1 (1.6-2.7) | 1 | 0.07 |
|  | Farming and Other | 27/1762 | 1.5 (1.1-2.2) | 0.84 (0.52-1.35) |  |
|  | Other, non-farming | 27/1268 | 2.1 (1.5-3.1) | 1.17 (0.73-1.87) |  |
|  | None | 50/1009 | 5.0 (3.8-6.5) | 1.54 (1.02-2.34) |  |
|  |  |  |  |  |  |
| Household Wealth tertiles | Lowest tertile | 62/2108 | 2.9 (2.3-3.8) | 1 | 0.08 |
|  | Middle tertile | 50/2120 | 2.4 (1.8-3.1) | 0.82 (0.57-1.20) |  |
|  | Highest tertile | 41/2185 | 1.9 (1.4-2.5) | 0.66 (0.44-0.97) |  |
|  |  |  |  |  |  |
| **SOCIAL** |  |  |  |  |  |
| Household size | 1 | 30/859 | 3.5 (2.4-5.0) | 1 | 0.09 |
|  | 2-5 | 86/3377 | 2.5 (2.1-3.1) | 0.87 (0.57-1.33) |  |
|  | 6+ | 37/2177 | 1.7 (1.2-2.3) | 0.61 (0.38-1.00) |  |
|  |  |  |  |  |  |
| Any social group participation | Yes | 43/3168 | 1.4 (1.0-1.8) | 1 | <0.001 |
|  | No | 110/3246 | 3.4 (2.8-4.1) | 1.94 (1.35-2.77) |  |
|  |  |  |  |  |  |
| Frequency of attending religious meetings | Never/almost | 110/3246 | 3.4 (2.8-4.1) | 1 | <0.001 |
|  | Once or twice a year | 14/426 | 3.3 (1.9-5.6) | 1.03 (0.59-1.80) |  |
|  | Every few months | 7 /492 | 1.4 (0.7-3.0) | 0.53 (0.25-1.14) |  |
|  | Once/twice a month | 22/2250 | 1.0 (0.6-1.5) | 0.38 (0.24-0.61) |  |
|  |  |  |  |  |  |
| Social support index | Low | 76/1854 | 4.1 (3.3-5.1) | 1 | <0.001 |
|  | Moderate | 54/2155 | 2.5 (1.9-3.3) | 0.77 (0.54-1.10) |  |
|  | High | 23/2405 | 1.0 (0.6-1.4) | 0.33 (0.20-0.53) |  |
| **PRIOR HEALTH CONDITIONS** | |  |  |  |  |
| Number of health conditions | None | 35/2148 | 1.6 (1.2-2.3) | 1 | 0.91 |
|  | One | 65/2547 | 2.6 (2.0-3.3) | 0.92 (0.60-1.44) |  |
|  | More than one | 53/1718 | 3.1 (2.4-4.0) | 0.99 (0.62-1.57) |  |
|  |  |  |  |  |  |
| HIV status | HIV Negative | 95/3234 | 2.9 (2.4-3.6) | 1 | 0.09 |
|  | HIV Positive | 58/3180 | 1.8 (1.4-2.4) | 1.36 (0.93-2.00) |  |
|  |  |  |  |  |  |
| Arthritis | Yes | 8 /263 | 3.0 (1.5-6.1) | 1 | 0.74 |
|  | No | 145/6150 | 2.4 (2.0-2.8) | 0.88 (0.43-1.81) |  |
|  |  |  |  |  |  |
| Chronic lung disease | Yes | 6 /253 | 2.4 (1.1-5.3) | 1 | 0.55 |
|  | No | 147/6161 | 2.4 (2.0-2.8) | 0.77 (0.34-1.75) |  |
|  |  |  |  |  |  |
| Stroke | Yes | 7 /149 | 4.7 (2.2-9.8) | 1 | 0.29 |
|  | No | 146/6264 | 2.3 (2.0-2.7) | 0.65 (0.30-1.39) |  |
|  |  |  |  |  |  |
| Angina | Yes | 4 /72 | 5.5 (2.1-14.8) | 1 | 0.24 |
|  | No | 149/6341 | 2.3 (2.0-2.8) | 0.52 (0.19-1.40) |  |
|  |  |  |  |  |  |
| Diabetes | Yes | 11 /328 | 3.4 (1.9-6.1) | 1 | 0.36 |
|  | No | 142/6086 | 2.3 (2.0-2.8) | 0.74 (0.40-1.37) |  |
|  |  |  |  |  |  |
| Hypertension | Yes | 57/2177 | 2.6 (2.0-3.4) | 1 | 0.91 |
|  | No | 96/4236 | 2.3 (1.9-2.8) | 0.98 (0.70-1.37) |  |
|  |  |  |  |  |  |
| **CLINICAL EXAMINATION** | |  |  |  |  |
| Hypertension level based on SBP and DBP* | Normal | 62/3464 | 1.8 (1.4-2.3) | 1 | 0.08 |
|  | Mild | 55/2124 | 2.6 (2.0-3.4) | 1.16 (0.81-1.68) |  |
|  | Grade 2/3 | 36/825 | 4.4 (3.1-6.0) | 1.63 (1.07-2.49) |  |
|  |  |  |  |  |  |
| Body Mass Index (kg/m^2^) | Underweight | 32/959 | 3.3 (2.4-4.7) | 1 | 0.37 |
|  | Normal | 80/3640 | 2.2 (1.8-2.7) | 0.79 (0.52-1.19) |  |
|  | Overweight | 32/1741 | 1.8 (1.3-2.6) | 0.70 (0.42-1.16) |  |
|  |  |  |  |  |  |
| **HEALTH RISK FACTORS** | |  |  |  |  |
| Tobacco use status | Never smoked | 106/4495 | 2.4 (1.9-2.9) | 1 | 0.17 |
|  | Stopped smoking | 26/982 | 2.6 (1.8-3.9) | 0.97 (0.61-1.53) |  |
|  | Currently smokes | 21/937 | 2.2 (1.5-3.4) | 0.64 (0.40-1.04) |  |
|  |  |  |  |  |  |
| Alcohol consumption | Never | 35/1807 | 1.9 (1.4-2.7) | 1 | 0.12 |
|  | Less than once a month | 73/3195 | 2.3 (1.8-2.9) | 1.29 (0.86-1.94) |  |
|  | At least once a month | 45/1411 | 3.2 (2.4-4.3) | 1.62 (1.02-2.56) |  |
|  |  |  |  |  |  |
| Hunger in the last 12 months | Never lacked food | 118/5090 | 2.3 (1.9-2.8) | 1 | 0.61 |
|  | Only some months | 20/744 | 2.7 (1.7-4.2) | 1.28 (0.80-2.06) |  |
|  | Almost every month | 15/579 | 2.6 (1.6-4.3) | 1.04 (0.61-1.79) |  |
|  |  |  |  |  |  |
| Health care access index | Good access | 24/1297 | 1.9 (1.2-2.8) | 1 | 0.36 |
|  | Moderate | 60/2763 | 2.2 (1.7-2.8) | 1.06 (0.66-1.71) |  |
|  | Poor access | 69/2353 | 2.9 (2.3-3.7) | 1.32 (0.82-2.11) |  |

**Supplementary Table 2: Mortality rates (per 100 person-years) and hazard ratios across broad types of disability adjusted by age, sex, and residence.**

| **Type of disability impairment** | **Number of deaths/Person-years of follow-up** | **Mortality Rate per 100 person-years** | **Hazard Ratio (95% CI) Age, sex, and location adjusted** | **Hazard Ratio (95% CI) Vision, Physical, and Psychological difficulties, Age, sex, and location adjusted** |
| --- | --- | --- | --- | --- |
|  |  |  |  |  |
| **OVERALL** | 153/6,414 | 2.4 (2.0-2.7) |  |  |
|  |  |  |  |  |
| **VISION difficulties** |  |  | ***p-value=0.011*** | ***p-value=0.063*** |
| Less than severe | 83/4,525 | 1.8 (1.5-2.3) | Reference | Reference |
| Severe/Extreme | 70/1,888 | 3.7 (2.9-4.7) | 1.53 (1.10-2.13) | 1.38 (0.98-1.94) |
|  |  |  |  |  |
| **PHYSICAL difficulties** |  |  | ***p-value=0.001*** | ***p-value=0.004*** |
| Less than severe | 45/3,432 | 1.3 (1.0-1.8) | Reference | Reference |
| Severe/Extreme | 108/2,982 | 3.6 (3.0-4.4) | 1.89 (1.30-2.74) | 1.77 (1.20-2.59) |
|  |  |  |  |  |
| **PSYCHOLOGICAL difficulties** |  |  | ***p-value=0.188*** | ***p-value=0.816*** |
| Less than severe | 100/4,774 | 2.1 (1.7-2.5) | Reference | Reference |
| Severe/Extreme | 53/1,639 | 3.2 (2.5-4.2) | 1.26 (0.89-1.78) | 1.04 (1.73-1.49) |
|  |  |  |  |  |

**Supplementary Table 3: Mortality rates (per 100 person-years) and hazard ratios across the changes in disability over the follow-up period adjusted by age and sex.**

| **Change in Disability over follow-up (based on WHODAS classification^1^)** | **Number of deaths/Person-years of follow-up** | **Mortality Rate per 100 person-years** | **Hazard Ratio (95% CI)Age and sex adjusted** |
| --- | --- | --- | --- |
|  |  |  |  |
| **OVERALL** | 153/6,414 | 2.4  (2.0-2.7) |  |
|  |  |  |  |
| **Persistently Non-severe Disability** | 76/4,689 | 1.6  (1.3-2.0) | Reference |
|  |  |  |  |
| **Improved Disability** | 2/253 | 0.8  (0.2-3.2) | 0.52  (0.13-2.09) |
|  |  |  |  |
| **Worsened Disability** | 25/978 | 2.6  (1.7-3.8) | 1.19  (0.74-1.91) |
|  |  |  |  |
| **Persistently Severe Disability** | 50/494 | 10.1  (7.7-13.4) | 3.45  (2.20-5.40) |
|  |  |  |  |
|  |  |  |  |
| Likelihood ratio test p-value |  |  | p<0.001 |

^1^WHODAS is WHO Disability Assessment Schedule.

**Supplementary Figure 4: Survival curves by WHODAS classification and sex of respondent**

| **a) In men**  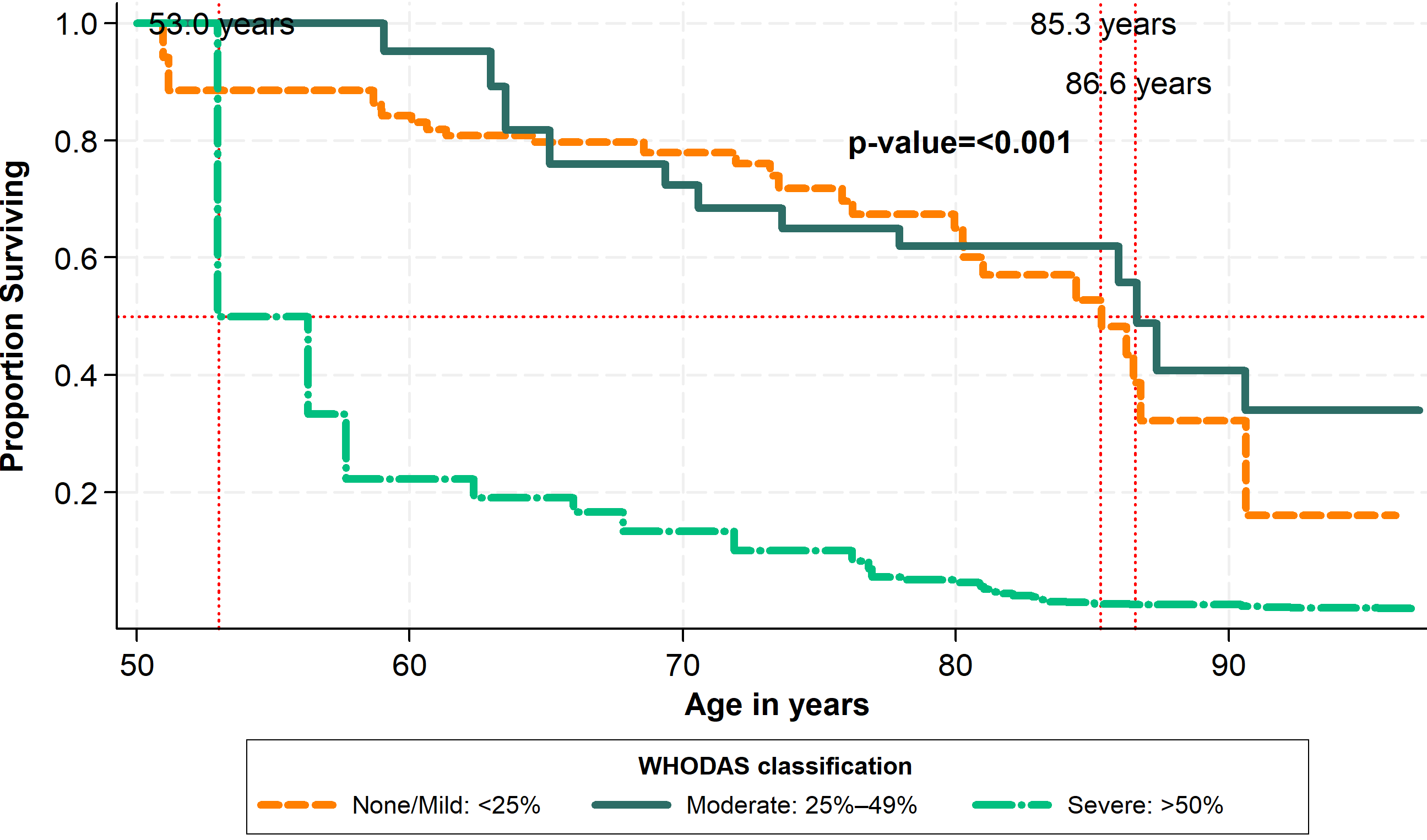 |
| --- |
| **b) In women**  **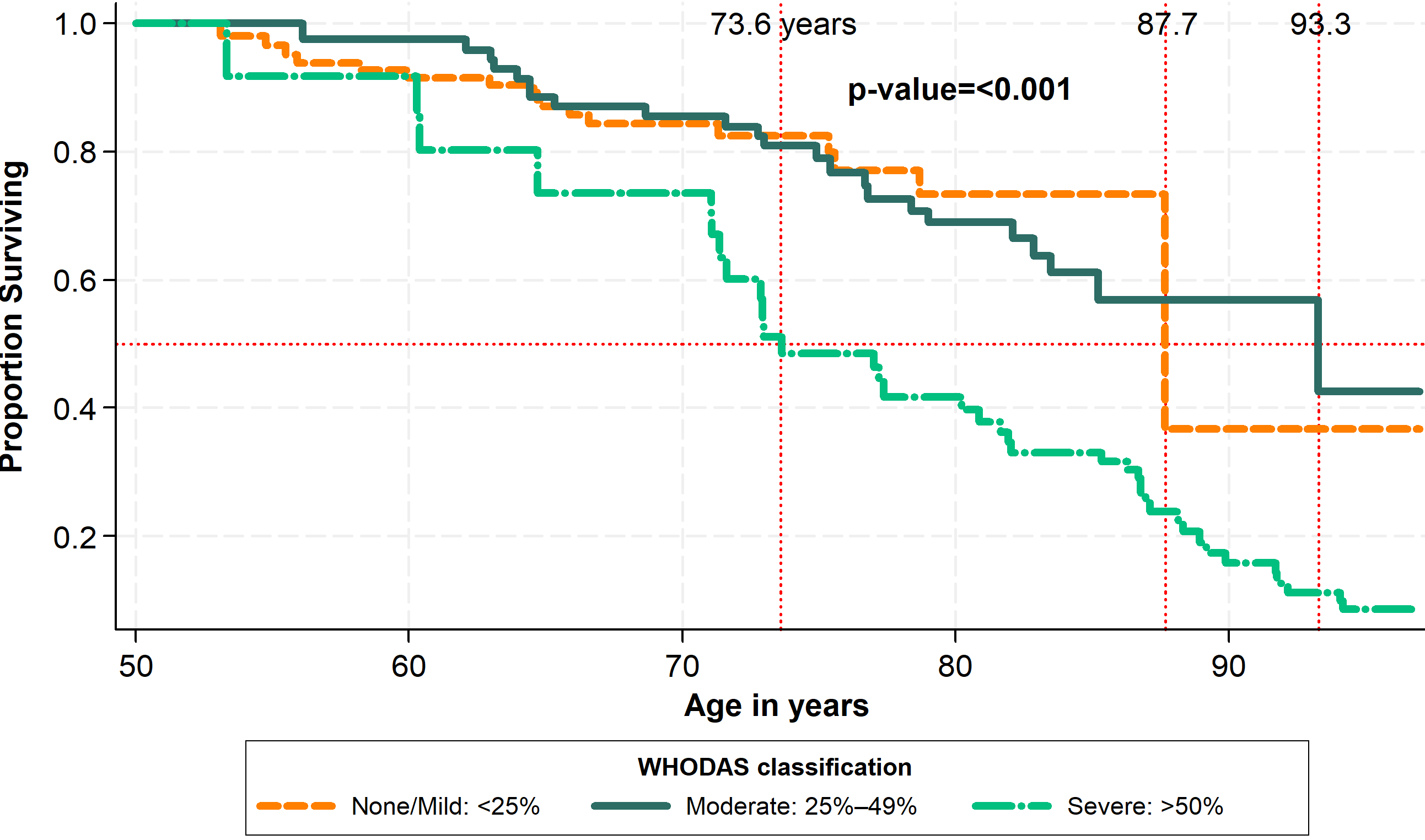** |

**Supplementary Figure 5: Cause of death categorization using verbal autopsy by sex**

**
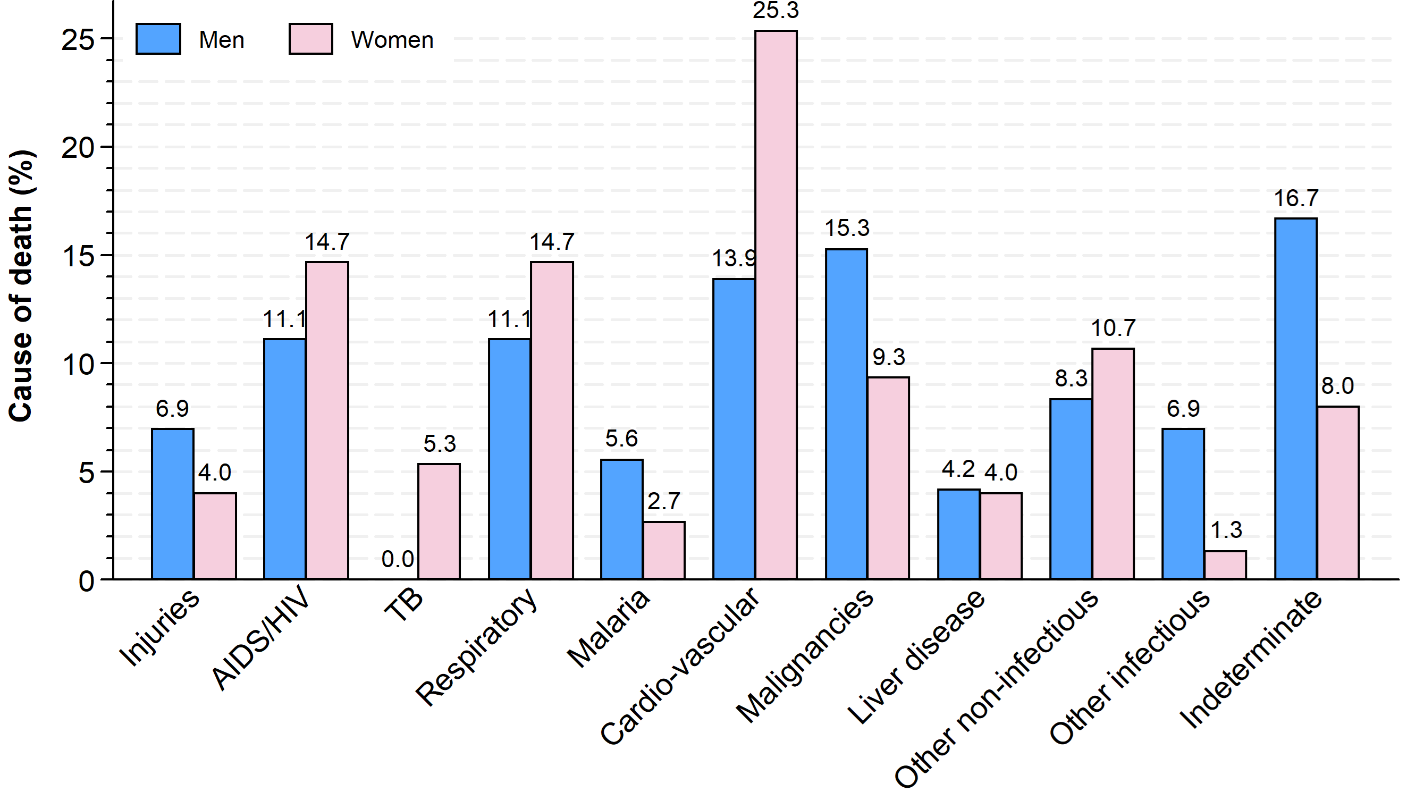
**
