# Supplementary material for: Association of disability and 12-year all-cause and cause-specific mortality: analyses from the Wellbeing of Older People cohort study in Uganda
Source: BMJ Glob Health. 2026 Jan 23;11(1):e019802. doi: 10.1136/bmjgh-2025-019802 (PMC12853530; doi:10.1136/bmjgh-2025-019802)
Supplement: online supplemental file 2 [file bmjgh-11-1-s002.docx]

### BMJ Global Health Author Reflexivity Statement

Adapted from Morton, B., Vercueil, A., Masekela, R., Heinz, E., Reimer, L., Saleh, S., Kalinga, C., Seekles, M., Biccard, B., Chakaya, J., Abimbola, S., Obasi, A. and Oriyo, N. (2022), Consensus statement on measures to promote equitable authorship in the publication of research from international partnerships. Anaesthesia, 77: 264-276. <https://doi.org/10.1111/anae.15597>

| **Study conceptualisation** | |
| --- | --- |
| 1. How does this study address local research and policy priorities? | This study was designed to examine the survival, health and wellbeing of older people. As such, it provides actionable data on informing the Uganda national strategies which emphasize Universal access to equitable, quality, age and disability-inclusive health services. This aligns with Uganda’s National Health Policy II and the Health Sector Development Plan. |
| 1. How were local researchers involved in study design? | The study co-designed and led by Principal Investigators at the MRC/UVRI and LSHTM Uganda Research Unit in collaboration with the World Health Organization. |
| **Research management** | |
| 1. How has funding been used to support the local research team(s)? | The funding supported the Ugandan research team including the co-investigators. The funding supported the salaries and research time of investigators, coordinators, data collectors, and analysts based at the MRC/UVRI and LSHTM Uganda Research Unit, as well as the field operations. Resources were also allocated for local training in quantitative and qualitative data, and research ethics, thereby strengthening national research capacity. All data management and analyses were conducted in Uganda, and local investigators led community engagement, stakeholder dissemination, and authorship of resulting publications. |
| **Data acquisition and analysis** | |
| 1. How are research staff who conducted data collection acknowledged? | The staff who led and conducted data collection have been included as co-authors on this manuscript. Pelegrino Mbabazi was the coordinator of the study. We also acknowledge the participants and the team which contributed to the collection of the data under the acknowledgement section. |
| 1. How have members of the research partnership been provided with access to study data? | The study data is widely available on secure and protected servers at the local institution. However, given that the study was multi-site and country, a copy of the data was also stored on a central location at the WHO portal accessible to researchers within and outside the partnership. |
| 1. How were data used to develop analytical skills within the partnership? | The local researchers and statisticians have led the analysis of the data. |
| **Data interpretation** | |
| 1. How have research partners collaborated in interpreting study data? | Data interpretation was carried out collaboratively through joint meetings (physical and virtual) between Ugandan and international partners. Local researchers led context-specific interpretation of findings to ensure analytical and policy relevance. |
| **Drafting and revising for intellectual content** | |
| 1. How were research partners supported to develop writing skills? | Local investigators and authors were supported through mentorship, and collaborative and iterative manuscript drafting to strengthen their scientific writing skills and ensure equitable authorship. |
| 1. How will research products be shared to address local needs? | The Ugandan investigators have led on the dissemination activities of the findings within Uganda through formal presentations to the research community and community dialogues. Research products will be disseminated in formats and through channels that meet local information and policy needs. Findings will be shared with the Uganda Ministry of Health, the Ministry of Gender, Labour and Social Development. |
| **Authorship** | |
| 1. How is the leadership, contribution and ownership of this work by LMIC researchers recognised within the authorship? | Ugandan investigators co-led the study and were central to its design, analysis, interpretation, and revisions. Therefore, these local researchers own and are recognised in the authorship reflecting their substantive intellectual contributions and ownership of the research. |
| 1. How have early career researchers across the partnership been included within the authorship team? | Early-career researchers from both the Ugandan and international teams led and were included as authors and mentored in data analysis, interpretation, presentation, and writing. |
| 1. How has gender balance been addressed within the authorship? | There was a fair gender balance in the authorship, including 3 males and 4 females. |
| **Training** | |
| 1. How has the project contributed to training of LMIC researchers? | The data collected from the projected is available and have been used by students on internships, and has been availed to researchers who have requested for the data to be used for MSc. courses. |
| **Infrastructure** | |
| 1. How has the project contributed to improvements in local infrastructure? | The project provided digital data collection tools, and computing facilities, and training and capacity building of Ugandan research staff at the MRC/UVRI and LSHTM Uganda Research Unit. |
| **Governance** | |
| 1. What safeguarding procedures were used to protect local study participants and researchers? | Safeguarding measures included ethical approval from UVRI Research and Ethical Committee (REC), Uganda National Council for Science and Technology, and LSHTM REC; informed consent in the main local language (Luganda); anonymised data handling; and referral procedures for unwell participants. Researchers received safeguarding and field safety training, consistent with national ethics guidelines. |
